# Recruitment patterns in a large international randomized controlled trial of perioperative care in cancer patients

**DOI:** 10.1186/s13063-021-05149-0

**Published:** 2021-03-20

**Authors:** Aaron Gazendam, Anthony Bozzo, Patricia Schneider, Victoria Giglio, David Wilson, Michelle Ghert

**Affiliations:** 1 Division of Orthopaedic Surgery, Department of Surgery, McMaster University, Hamilton, Ontario Canada; 2 Centre of Evidence-Based Orthopaedics, McMaster University, Hamilton, Ontario Canada

**Keywords:** Orthopedic oncology, Surgical, Randomized controlled trial, Recruitment

## Abstract

**Introduction:**

The Prophylactic Antibiotic Regimens in Tumor Surgery (PARITY) randomized controlled trial (RCT) was the first study to prospectively enroll and randomize orthopedic oncology patients in multiple centers internationally. The objective of this study was to describe recruitment patterns, to examine the differences in enrollment across different PARITY sites, and to identify variables associated with differing levels of recruitment.

**Methods:**

Data from this study was obtained from the PARITY trial Methods Center and records of correspondence between the Methods Center and recruiting sites. We performed descriptive statistics to report the recruitment patterns over time. We compared recruitment, time to set up, and time to enroll the first patient between North American and international sites, private and public healthcare models, and the presence or absence of research personnel. Two-tailed non-paired *t* tests were performed to test average monthly recruitment rates between groups.

**Results:**

A total of 602 patients from 36 North American and 12 international sites were recruited from 2013 to 2019. North American sites were able to become fully enrollment-ready at an average of 19.5 months and international sites at an average of 27 months. Once enrolling, international sites were able to enroll 0.59 patients per/month whereas North American sites averaged a monthly recruitment rate of 0.2 patients/month once enrolling. Sites with research personnel reached enrollment-ready status at an average of 19.3 months and sites without research support at an average of 30.3 months. Once enrolling, the recruitment rate was 0.28 patients/month and 0.2 patients per month for sites with and without research support, respectively. Publicly funded sites had a monthly enrollment of 0.4 patients/month whereas privately funded sites had a monthly enrollment rate of 0.17 patients/month.

**Conclusions:**

As a collaborative group, the PARITY investigators increased the pace of recruitment throughout the trial, likely by increasing the number of active sites. The longer time to start-up at international sites may be due to the complex governing regulations of pharmaceutical trials. Nevertheless, international sites should be considered essential as they recruited significantly more patients per month once active. The absence of research support personnel may lead to delays in the time to start-up. The results of the current study will provide guidance for choosing which sites to recruit for participation in future collaborative clinical trials in orthopedic oncology and other surgical specialties.

**Trial registration:**

ClinicalTrials.gov NCT01479283. Prospectively registered on November 24, 2011

## Introduction

Randomized controlled trials (RCTs) are widely recognized as the study design that can provide the highest level of clinical effectiveness evidence with the minimal amount of systematic bias [1]. When compared to the medical field, surgical specialties often lack high-quality RCT evidence on which to base clinical decisions [2, 3]. Surgically based RCTs present with a unique set of challenges that can lead to systematic bias such as lack of blinding, surgeon preferences, poor patient compliance, and loss to follow-up [3–5].

In order to reach the target sample size and to increase the generalizability of results, RCTs are often multicenter and international [6]. However, international trials are burdened with significant challenges such as differences in regulatory bodies, varying sources of funding, protocol standardization, language barriers, and language translation errors [7]. These challenges can make timely recruitment and enrollment difficult to achieve. It is well documented that a significant number of clinical trials fail to meet enrollment goals, which may lead to underpowered studies and imprecise findings, extended study duration, or even trial abandonment [8–12]. A variety of site-specific variables have been found to impact enrollment performance in clinical trials including patient volume, previous clinical research involvement, lead investigator enthusiasm, and dedicated support staff [13–16].

Within the surgical subspecialty of orthopedic oncology, there is very little high-level evidence to inform clinical decision-making—including a lack of international multicenter surgical RCTs [17, 18]. Given the rarity of soft tissue and bone sarcomas, a collaborative multicenter approach is needed. However, due to the extreme heterogeneity of both case complexity as well as treatment protocols, trials in orthopedic oncology represent a unique challenge in RCT implementation. The Prophylactic Antibiotic Regimens in Tumor Surgery (PARITY) trial was the first international multicenter trial in orthopedic oncology and aimed to provide high-quality evidence for prophylactic antibiotic therapy in patients undergoing surgical management for lower extremity bone tumors [19, 20]. The trial completed enrollment in 2019. Given that this was the first RCT of its kind in the field, it is important to reflect upon the challenges encountered and experiences gained in order to inform future trials. The objective of this study was to describe recruitment patterns, to examine the differences in enrollment across different PARITY sites, and to identify variables associated with differing levels of recruitment.

## Materials and methods

### PARITY trial

Data for this study was derived from the PARITY trial (NCT01479283), the protocol for which has been previously published [19]. PARITY was a blinded, parallel two-arm multicenter international RCT. Study enrollment began in January 2013 and was completed in October 2019. Target enrollment was 600 participants based on a *pre hoc* sample size calculation. Inclusion criteria consisted of patients 12 years of age or older who had a lower extremity primary bone malignancy, a benign aggressive bone tumor, a soft-tissue sarcoma which had invaded the bone, or oligometastatic bone disease and required surgical excision and endoprosthetic reconstruction of the femur and/or tibia were included.

Study participants were randomized to either short- (24 h) or long-duration (5 days) post-operative prophylactic antibiotics. A centralized, Internet-based computerized randomization system was utilized to conceal allocation. The primary outcome for the trial was surgical site infection.

### Recruitment patterns

We obtained several variables that have been previously associated with differing levels of recruitment in RCTs directly from local site investigators [21–24]. We compared recruitment levels between North American and international sites, private or public healthcare models, and the presence or absence of research personnel. We defined the presence of research personnel as a dedicated research nurse, coordinator, or assistant available to assist the local Principal Investigator in the conduct of the trial. Differentiating between North American and international sites was undertaken for a number of reasons. This trial was funded through the Musculoskeletal Tumor Society, a largely North American-based society, making North American sites the initial focus of the trial. The decision to add international sites was based on the growing international interest in collaboration. Given that the majority of these sites operated in languages outside of the English language, they were considered operationally different with unique language-based challenges [25].

We defined the rate of recruitment as patients recruited per month. We defined the time to set-up (TTSU) as the time from when an interested site first contacted the PARITY Methods Center to the time when the site was deemed able to begin enrolling—after having secured ethics approval, completed training, and signed all clinical trial agreements. We defined time to first patient (TTFP) as the interval between when a site was ready to enroll patients and when the first patient was enrolled at that site.

### Statistical methods

We performed descriptive statistics to evaluate the recruitment patterns over time. We used two-tailed non-paired *t* tests to explore differences in average monthly recruitment rates between North American and international sites, between private or public healthcare systems, by the presence or absence of dedicated research personnel, and if monthly enrollment varied by TTSU or TTFP. We also compared TTSU and TTFP based on location (North America vs international), healthcare funding model, and research personnel. We also calculated the Pearson *R* correlation coefficient between TTSU, TTFP, and average enrollment rate. We used the chi-square test to assess for the relationship between categorical variables, when applicable. Significance for all tests was set at alpha = 0.05. We performed all analyses in Microsoft Excel [26].

## Results

### Site characteristics

**Fig. 1:**
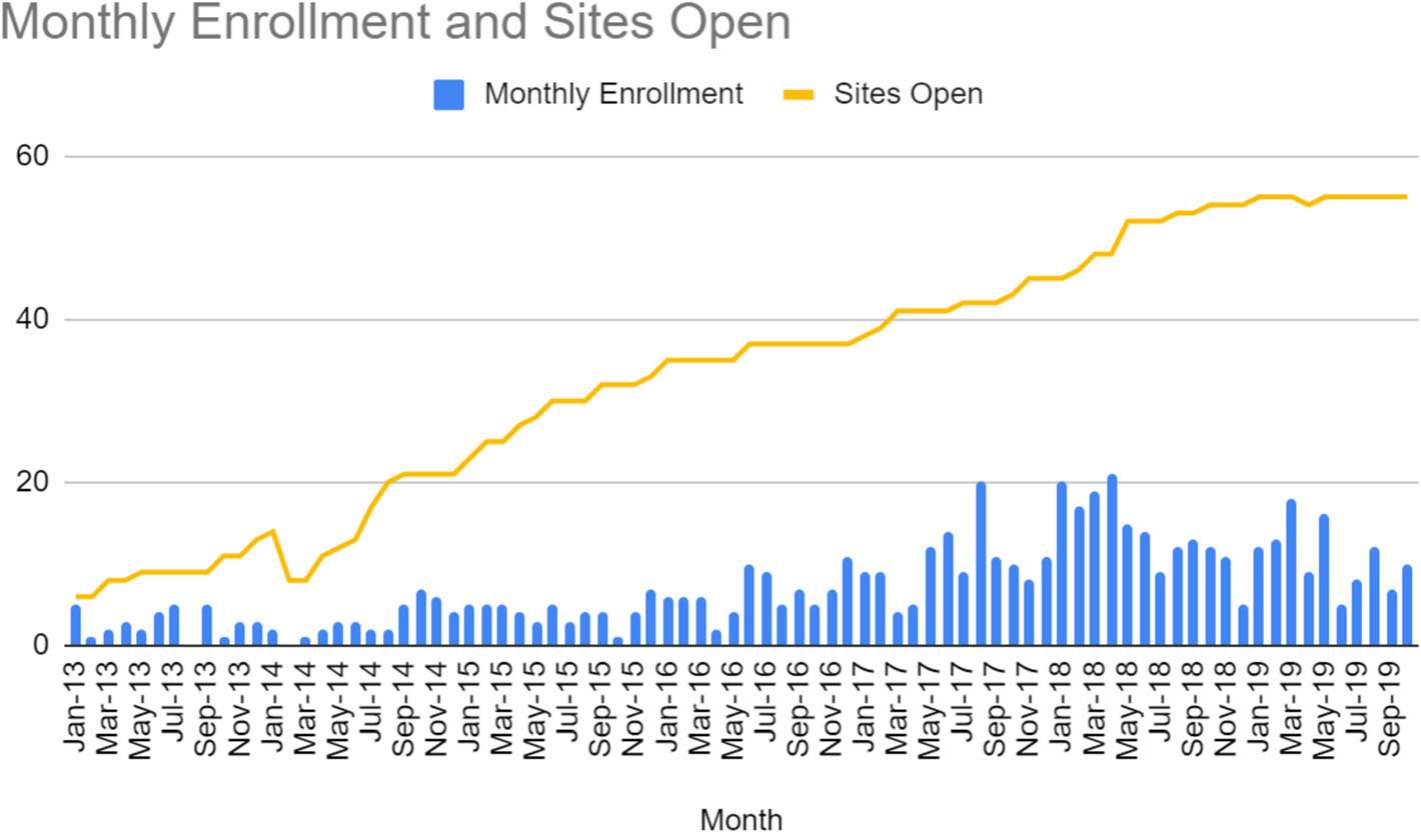
Monthly enrollment and sites open for each month of the study period

A total of 55 sites obtained ethics approval and signed clinical trial agreements. Forty-eight (87%) of these open sites recruited at least one patient during the study period and were deemed active, while seven sites did not enroll any patients in the study. There were 44 other sites that expressed interest and/or received study contracts but never completed the process of becoming an open site. Table 1 and Fig. 1 demonstrate the number of active sites and patients enrolled per month over the study period. A total of 36 active sites were North American and 12 were international. When characterized by the healthcare model, 19 sites were predominately publicly funded and the remaining 29 were predominately privately funded. Of the privately funded sites, 28 were based in the USA and one site was based in Argentina.

**Table 1:** Average recruitment over the study period

Year	Average monthly enrollment	Open sites
**2013**	2.8	13
**2014**	3.1	20
**2015**	4.2	33
**2016**	6.5	37
**2017**	10.2	45
**2018**	14.0	54
**2019**	11.0	55

### Patient enrollment rate

A total of 602 patients from 48 clinical sites in 12 countries were recruited from January 2013 through to October 2019. While the average number of patients enrolled throughout the study was 7.3 patients/month (SD ± 5.2), average monthly enrollment increased every year until the last year of the study and enrollment was greater than 10 patients/month in 2017, 2018, and 2019 (Table 1 and Fig. 1). While 58 months were required to reach 50% of target enrollment (January 2013 to October 2017), only an additional 24 months was required to achieve 100% enrollment. The top 20% of sites in terms of patient enrollment accounted for almost 60% of all included patients (355/602). The highest enrolling single site for the trial accounted for 14% of all patients (84/602).

### Site opening and enrollment

**Fig. 2:**
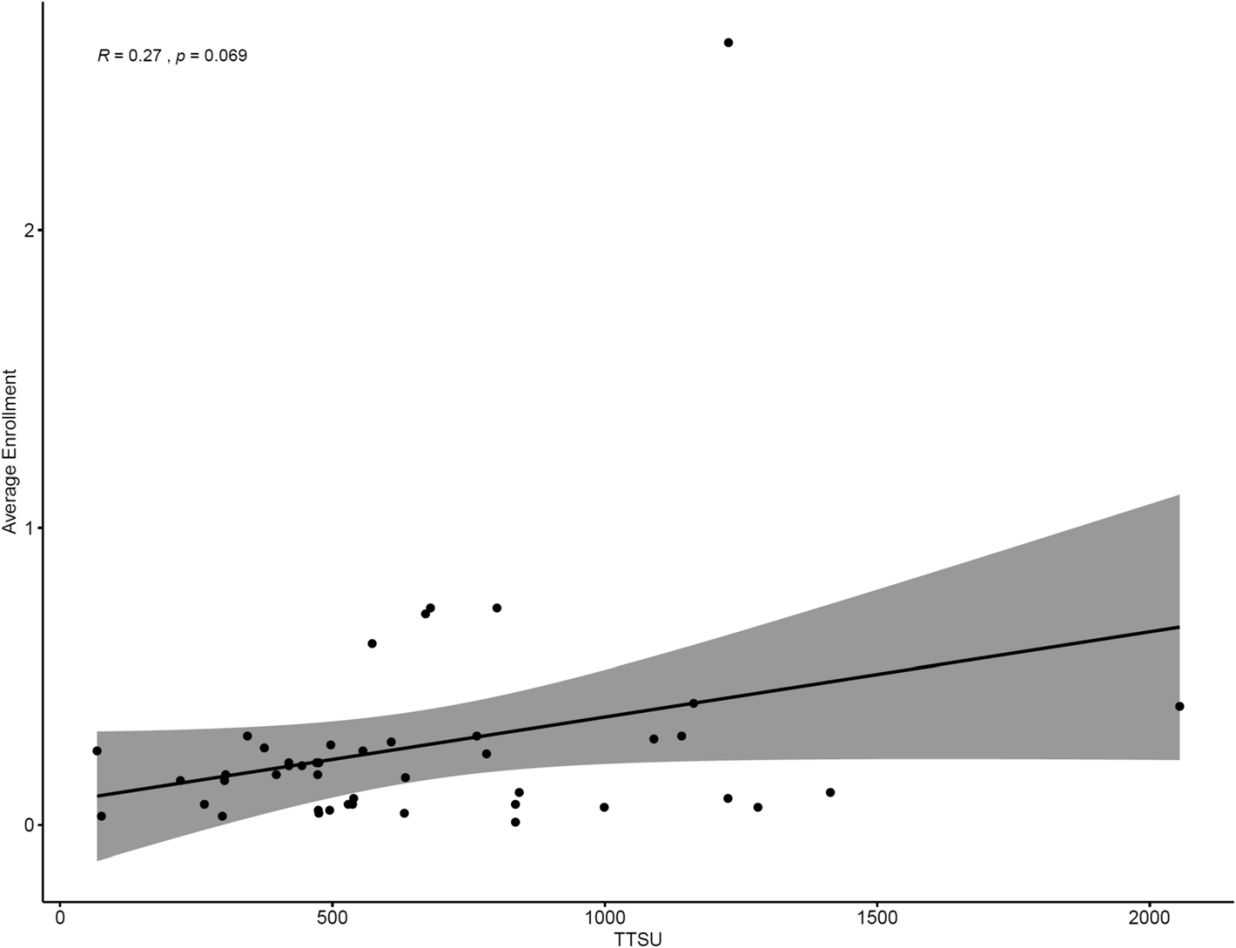
TTSU vs enrollment rate. Sites with faster TTSU did not display higher enrollment rates. No significant correlation is observed between TTSU and average enrollment (Pearson *R* = .27, *p* = 0.069)

There was no correlation between TTSU and the site’s average monthly enrollment (Pearson *R* = 0.27, *p* = 0.069) (Fig. 2). However, once set up, the time needed for a site to recruit their first patient (TTFP) was predictive of the number of total patients recruited and the average recruitment rate of that site. Generally, the time needed to recruit the first patient was inversely correlated to the average monthly enrollment rate of that site (Pearson *R* = − 0.44, *p* = 0.0033). In fact, sites that required longer than 1 year to recruit their first patient had an average recruitment rate three and a half times lower than that of sites that were able to recruit their first patient within 1 year (0.09/month vs 0.32/month, *p* = 0.04).

### Site location and healthcare system

There were 36 (75%) North American and 12 (25%) international active sites that enrolled patients. North American sites were able to become fully enrollment-ready at an average of 19.5 months and international sites at an average of 27 months. Once enrolling, international sites were able to enroll 0.59 patients per/month whereas North American sites averaged a monthly recruitment rate of 0.2 patients/month once enrolling (Table 2). The TTFP of North American and international sites was 8.7 and 4.5 months, respectively. The proportion of sites that demonstrated interest but did not become active or recruit a single patient was 25/61 and 26/38 for international and North American sites, respectively. When considering the healthcare model, the TTSU was 20.1 months in privately funded sites and 23.4 months in publicly funded sites. Publicly funded sites had a monthly enrollment of 0.4 patients/month whereas privately funded sites had a monthly enrollment rate of 0.17 patients/month.

**Table 2:** Enrollment patterns

**Group**	*Enrollment (patients/month ± SD)*	***p*** **value**
**North American (36)**	0.20 ± 0.18	0.023^a^
**International (12)**	0.59 ± 0.73	
**Public/mixed (19)**	0.40 ± 0.56	0.03^a^
**Private (29)**	0.17 ± 0.14	
**Research personnel (40)**	0.28 ± 0.42	0.63
**No research personnel (8)**	0.2 ± 0.16	
**Group**	*Time to start-up (months ± SD)*	*p* value
**North American (36)**	19.5 ± 9.0	0.04^a^
**International (12)**	27.0 ± 19.5	
**Public/mixed (19)**	23.4 ± 16.5	0.39
**Private (29)**	20.1 ± 9.4	
**Research personnel (40)**	19.3 ± 9.6	0.03^a^
**No research personnel (8)**	30.3 ± 22.3	
**Group (*n*)**	*Time to first patient (months ± SD)*	*p* value
**North American (36)**	8.7 ± 11.9	0.25
**International (12)**	4.5 ± 5.1	
**Public/mixed (19)**	6.9 ± 15.0	0.7
**Private (29)**	8.1 ± 7.02	
**Research personnel (40)**	8.0 ± 11.3	0.62
**No research personnel (8)**	5.9 ± 7.3	

Two-tailed non-paired *t* tests were used to test the statistical significance

*SD* standard deviation

^a^Statistical significance with alpha = 0.05

### Research personnel

Of active enrolling sites, 40 (83%) had research support personnel and 8 (17%) sites did not. Sites with research personnel reached enrollment-ready status at an average of 19.3 months and sites without research support at an average of 30.3 months. Once enrolling, the recruitment rate was 0.28 patients/month and 0.2 patients per month for sites with and without research support, respectively.

## Discussion

The PARITY trial is the first multicenter surgical RCT in orthopedic oncology and successfully achieved target recruitment. Increasing the number of open sites each year led to the continually increasing monthly enrollment rates, which was crucial to reaching target enrollment in a reasonable time frame. Recruitment of the second half of the patient cohort required less than 50% of the time required to recruit the first half (24 vs 58 months). While international sites required more time to become enrollment-ready (TTSU), a higher average monthly enrollment was observed at these sites once they were active. Sites without dedicated research personnel also demonstrated a longer TTSU, but enrolled at equivalent rates once active. Whether a site was publicly or privately funded did not affect TTSU; however, recruitment rates were faster in publicly funded sites. Sites that took longer than 1 year to recruit their first patient once active were unlikely to recover their recruitment rate thereafter and demonstrated an average recruitment rate of less than one third that of sites that were quicker to enroll.

Compared to medical trials, surgical trials have high rates of failure and are often discontinued due to slow recruitment [27, 28]. Incomplete or under-recruited RCTs pose significant problems through the waste of public funding and the reporting of inaccurate results [29, 30]. The presence of trial fatigue can slow recruitment and decrease the likelihood that a trial will be adequately powered [15]. The PARITY Methods Center employed a number of strategies to maintain a high trial profile to encourage ongoing recruitment throughout the recruitment phase of the trial. Attending and presenting updates at national and international conferences allowed co-investigators to stay engaged in the trial. The trial website was a platform that allowed all investigators to remain up to date on the trial progress (www.PARITYtrial.com). The website also highlighted new sites and invited all potential investigators to join the trial. The PARITY team also delivered several newsletters annually via email to keep investigators up to date on trial progress. These strategies have been employed widely in RCTs in the past; however, due to the heterogeneity of recruitment strategies utilized, no formal comparisons to assess their efficacy have been undertaken [31]. Although we were unable to quantify the effects of these interventions, the PARITY investigators felt that these interventions were important in increasing the year-by-year enrollment over the duration of the study.

International trials present unique challenges and benefits [6]. Although they have the potential to increase recruitment and provide generalizable data, international trials are also burdened by increased regulatory burden, data quality, safety monitoring, and language barriers [25, 32–34]. The PARITY trial demonstrated that international sites outside of North America took significantly longer to achieve regulatory approval. In addition, it was more likely for international sites to fail to become enrollment-ready. However, once approved, international sites contributed significantly to recruitment. Delays in regulatory approvals in international trials are not unique to PARITY and have been encountered by several previous RCTs [21, 22]. Aban et al. conducted a US-based international trial determining the efficacy of steroids in Duchenne-muscular dystrophy. The authors demonstrated longer start-up times by international sites and cited delays in ethics approval, language barriers, and varying regulatory bodies as potential causes [22]. Similarly, Crow et al. cited language barriers and the need for translation of key study documents prior to submission to local ethics committees as a major unintended financial cost and delay in site start-up in a multinational RCT [35].

We have decided not to publish the list of contributing sites, as requested by the reviewer, until the PARITY trial results themselves are published in 2021. The publication of this data would violate the proprietary rights of the PARITY investigators who contributed to the trial but did not agree to have identifiable individual site data published outside of the trial itself. In addition, the identity of the sites and investigators are beyond the scope of the current study.

Our findings also suggest ongoing challenges with clinical trial recruitment in insurance-based healthcare systems. It is possible that the lower volume of patients seen in a privatized healthcare system is due to the lack of centralized care. Some private hospitals have a single orthopedic oncologist and significantly lower volume than publicly funded hospitals that provide centralized care for a larger population and therefore treat a significantly higher volume of patients. The resources required to start up low-volume sites can result in recruitment delays, increased start-up costs, and increased yearly funding required to keep a large number of low-volume sites open. Alternatively, lower recruitment in privatized healthcare centers may have been due to a lack of insurance coverage for participation. Klamerus et al. demonstrated that 13.6% of patients consented for cancer clinical trials were unable to participate due to lack of insurance coverage [23].

Clinical research coordinators and research assistants are considered integral to running and completing a successful clinical trial [36–38]. Survey-based research has determined that the majority of Principal Investigators believe that clinical research personnel are able to improve recruitment [39]. Results from our study demonstrate that sites with research personnel were able to start up significantly faster than sites without research personnel. However, once active, there was no difference in the monthly recruitment between sites that had research personnel and those that did not. A lack of difference in recruitment may have been secondary to reasons unrelated to research personnel such as low patient volumes or challenges with insurance companies. Based on our findings, we conclude that in orthopedic oncology research personnel are valuable and significantly improve trial efficiency by speeding up site start-up time.

A major strength of this study is the novelty of the findings. This is the first paper to our knowledge to examine variables associated with varying rates of recruitment in RCTs within orthopedic surgery. It provides important information about expected challenges and patterns of recruitment for future RCTs in the field. A limitation of the current paper is that the underlying reasons for differing levels of recruitment could not be fully explored with the available data. Although we can make hypotheses about differences among sites, without in-depth interviews with the site investigators, we cannot make conclusions about site-specific facilitators or barriers to successful recruitment. Future considerations for qualitative analysis of interviews with local principal investigators will allow for a deeper understanding of causative factors of varying levels of recruitment.

## Conclusions

As a collaborative group, the PARITY investigators increased the pace of recruitment throughout the trial. The longer time to start-up at international sites is likely due to the complex governing regulations of pharmaceutical trials. Nevertheless, international sites should be considered essential as they recruited significantly more patients per month once active. The PARITY trial also highlights the ongoing challenges with recruitment in insurance-based healthcare systems. The absence of research support personnel is likely to result in delays in the time to start-up. The results of the current study provide important information to guide participation and recruitment in future collaborative clinical trials in orthopedic oncology and other surgical specialties.

## Data Availability

The data sets used and analyzed during the current study are available from the corresponding author on reasonable request.
